# Novel applications of liquid Biopsy: Comprehensive methodology for circulating biomarker exploration in peripheral blood

**DOI:** 10.1016/j.jlb.2025.100307

**Published:** 2025-06-26

**Authors:** Caterina De Rosa, Luisa Amato, Annalisa Ariano, Sara Capaldo, Daniela Esposito, Hamid Heydari Sheikhhossein, Alessia Salzillo, Alessandra Di Liello, Concetta Tuccillo, Martina Cesarano, Daniela Frezzetti, Rosa Carmelingo, Antonella De Luca, Antonio Gambardella, Virginia Tirino, Fortunato Ciardiello, Floriana Morgillo, Federica Papaccio, Alberto Servetto, Viviana De Rosa, Francesca Iommelli, Carminia Maria Della Corte

**Affiliations:** a Department of Precision Medicine, University of Campania Luigi Vanvitelli, 80131, Naples, Italy; b Department of Clinical Medicine and Surgery, University of Naples Federico II, 80131, Naples, Italy; c Department of Medical, Oral and Biotechnological Sciences, University “G. d’Annunzio” of Chieti-Pescara, 66013, Chieti, Italy; d Villa Serena Foundation for Research, 65013, Città Sant'Angelo, Italy; e Cell Biology and Biotherapy Unit, Istituto Nazionale Tumori-IRCCS-Fondazione G. Pascale, 80131, Naples, Italy; f Department of Experimental Medicine, University of Campania Luigi Vanvitelli, 81100, Caserta, Italy; g Department of Medicine, Surgery and Dentistry, “Scuola Medica Salernitana”, University of Salerno, 84081, Baronissi, Italy; h Clinical Pharmacology Unit, San Giovanni di Dio e Ruggi d’Aragona University Hospital, 84131, Salerno, Italy; i Institute of Biostructures and Bioimaging, National Research Council, 80145, Naples, Italy

**Keywords:** Circulating tumor cells, Liquid biopsy, Exosomes, Peripheral blood immune cells, Tumor-educated platelets

## Abstract

The liquid biopsy (LB) represents a minimally invasive method for cancer screening that has been introduced in clinical practice for over a decade and that can accelerate treatment response assessment. LB allows the analysis of tumor cells or tumor-derived products (e.g. cell-free circulating nucleic acids, extracellular vesicles, and proteins) released from primary or metastatic tumor lesions into blood or other body fluids. In the era of immune-oncology, recent evidence indicates that tumor-specific immune responses can be detected in peripheral immune cells. The improvement of knowledge and the standardization of the isolation methods of these techniques will allow the detection and characterization of circulating tumor and immune biomarkers at an early stage as innovative tools to predict response to therapies. Nowadays, the analysis of peripheral blood mononuclear cells (PBMCs), circulating tumor cells (CTCs), peripheral blood-derived extracellular vesicles (EVs) and circulating tumor RNA (ctRNA) remains under-developed even if these non-invasive techniques can provide the complete genetic landscape of tumors and allow systematic tracking of cancer evolution. In addition, the evaluation of blood circulating cytokines, and early dynamics changes in the PBMCs of patients with solid tumors represent a promising area of research. Here, we present a comprehensive methodological framework for the evaluation of innovative peripheral blood-derived biomarkers. We also address the current challenges in isolation methods and analysis of PBMC, CTC, EVs and TEPs which are crucial for structuring the large amount of comprehensive information obtained from such samples, with the aim of advancing the translational cancer field.

## Highlights

• Comprehensive detailed protocols for liquid biopsy applications.
• Address the current challenges in isolation methods of circulating blood cells.
• Reduces the gap due to the lack of standardized protocols.
• Aims to advance the translational cancer field.

## Graphical abstract

**Figure:**
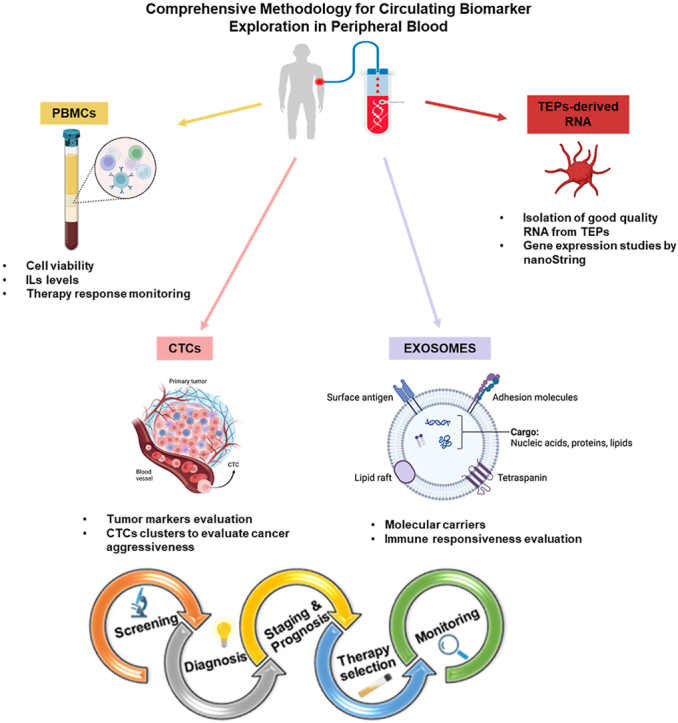

## 1 Introduction

In the era of precision medicine, molecular profiling is essential since it may transform the way cancer is diagnosed and treated. Liquid biopsy (LB) has emerged as a particularly impactful tool, having the potential to overcome many of the challenges associated with traditional histopathological tissue biopsies, such as intra-tumor heterogeneity and tumor evolution over time, particularly in cases where tumor tissue is unavailable or insufficient, such as in lung cancers [1], with potential clinical applications of LB in lung cancer such as screening, monitoring residual disease and treatment efficacy and predicting tumor evolution [2]. Clinical utility remains still unproven in most contexts, since we still lack data from prospective studies. First introduced in 2010 for circulating tumor cells (CTCs), LB provides a real-time information on circulating cell-free tumor DNA (ctDNA), circulating cell-free RNA (non-coding, miRNA and messenger RNA), extracellular vesicles (EVs) (exosomes and oncosomes), tumor-derived platelets, circulating proteins, immune cells and immune system components [3]. In particular, some new biomarkers are emerging, such as: the gene expression profile of peripheral blood mononuclear cells (PBMCs) [4] and of cancer-derived types of RNA reposited in tumor-educated platelets (TEPs) [5]. Similarly, exosomes are a subset of EVs, players in intercellular communication delivering a payload of molecular cargo [6], with potential as novel biomarkers from LB, in cancer.

In this regard, standardized technical procedures are foundational to translating LB biomarkers into clinically actionable tools. Variability in pre-analytical and analytical workflows—such as sample collection, storage, and processing—can introduce significant bias, compromising the reproducibility of results across studies. For instance, inconsistencies in blood draw volumes, anticoagulant use, or delays in plasma separation may alter the quantity and quality of ctDNA or EVs. By adopting harmonized protocols, clinicians can ensure reliable biomarker quantification, enabling precise risk stratification (e.g., minimal residual disease detection) and therapy selection (e.g., targeting EGFR mutations in NSCLC). Standardization may also facilitate multicenter collaborations, which are critical for validating LB biomarkers in diverse patient cohorts and integrating them into consensus guidelines like RECIST or iRECIST.

Here, we present a comprehensive methodological framework for the evaluation of innovative peripheral blood-derived biomarkers, describing new protocols and methodological approaches.

## 2 Materials and methods

### 2.1 cfDNA isolation procedures

Although cfDNA analysis is not among the focus of this manuscript, we report the main methodologies currently employed for cfDNA isolation. Indeed, cfDNA represents a cornerstone of LB, with FDA/EMA-approved assays relying on silica-membrane columns or magnetic bead-based extraction [1]. Regulatory-approved assays and clinical applications include FoundationOne® Liquid CDx, an FDA-approved test that detects mutations in 311 genes to guide therapy selection for NSCLC, prostate, and breast cancers. The FDA-approved Guardant360® investigates genomic alterations in 73 cancer-related genes. The cobas® EGFR Mutation Test v2, FDA- and EMA-approved, is a PCR-based companion diagnostic for osimertinib in NSCLC, optimized for EDTA-treated plasma. These assays utilize silica-membrane columns, such as the QIAamp Circulating Nucleic Acid Kit, or magnetic bead-based extraction, including the MagMax Cell-Free DNA Kit, with recovery rates varying by fragment size, typically 50–70 % for 160–180 bp fragments [3]. However, clinical outcomes are influenced by pre-analytical variables: EDTA vs. Streck tubes for blood collection impact cfDNA yield, while centrifugation speeds (e.g., 1600–3000×*g*) affect cellular DNA contamination (Table 1).

**Table 1:** Pre-Analytical Workflow Standardization for critical variables influencing cfDNA yield and quality.

Variable	Impact	Mitigation Strategy
Blood collection	EDTA tubes → leukocyte lysis ↑; Streck/PAXgene® tubes → cfDNA stability ↑	Use cell-stabilizing tubes for delays >6h.
Centrifugation	1st spin (1600×*g*): Removes cells; 2nd spin (16,000×*g*): Clears debris.	Standardize spins to avoid genomic DNA contamination.
Storage	Plasma stored >24h at 4 °C → cfDNA degradation ↑.	Freeze plasma at −80 °C in single-use aliquots.

Commercial kits (QIAamp Circulating Nucleic Acid Kit, MagMax Cell-Free DNA) vary in recovery rates for short (<150 bp) vs. long fragments, skewing tumor-derived ctDNA detection. Standardizing these steps is critical, as discordant protocols may lead to false-negative results in low-abundance mutations. In response to these challenges, several emerging solutions are being implemented to enhance the standardization and reliability of cfDNA isolation. The use of synthetic reference materials, such as Horizon Discovery's ctDNA controls containing defined tumor fractions, allows for the calibration and benchmarking of next-generation sequencing (NGS) assays across laboratories. Automation of extraction workflows, such as QIAsymphony® platforms has been shown to reduce inter-operator variability, which is particularly beneficial in high-throughput clinical settings. Furthermore, analytical techniques such as fragment size analysis using instruments like the Agilent 4200 TapeStation enable the verification of cfDNA enrichment for tumor-derived fragments, typically peaking around 166 base pairs, thereby ensuring the quality and consistency of the isolated material.

### 2.2 PBMCs isolation with density gradients methods

Since 1968, Ficoll-Paque density gradient centrifugation remains a standard PBMC isolation method. However, it requires optimization to enhance yield, consistency, and quality. We present an optimized protocol for PBMC extraction from limited blood volumes (Diagrammatic Representation 1).

#### 2.2.1 Materials and equipment

● Burker cell counter (Sigma-Aldrich, St. Louis, MO, USA; cat. n. Z359629)
● Coverslip (Sigma-Aldrich, St. Louis, MO, USA; cat. n. Z375357).
● Dulbecco's Phosphate buffered saline D-PBS (Gibco™, cat. no. 14190144).
● Centrifuge (Thermo Scientific, Cincinnati, OH, USA; model Heraeus Megafuge 1.0).

**▲ NOTE: The centrifuges must be equipped with a circuit breaker to turn off the brake.**

● Class II biological safety cabinet (Steril, Lecce, Italy; model. n. VBH 48 MP VBH).
● EDTA tubes (Fisher Scientific, Pittsburgh, PA, USA; model BD 367862).
● Flow Cytometer (optional).
● Humidified 95 % O2, 5 % CO2 water jacketed incubator, 37 °C (Thermo Scientific, Cincinnati, OH, USA; model Forma Series II) (optional).
● Inverted microscope (Leica Microsystems, Milan, Italy; model DMi1).
● Lymphocyte Separation Medium (Lonza, Walkersville, MD, USA; cat. n. 17-829E), or Lymphoprep™ (StemCell Technologies, cat. no. 07811).
● Pipettes (Eppendorf Research, Milan, Italy; cat. n. 3123000020 (0.5–10 μL), n. 3123000047 (10–100 μL), n. 3123000063 (100–1000 μL), with relative sterile tips).
● RBC Lysis Buffer (Invitrogen™, Waltham, MA, USA, cat. no. 00-4333-57).
● Trypan blue (Sigma Aldrich, St Louis, MO, USA; cat. n. T8154).
● 1.5 mL tube (Eppendorf Research, Milan, Italy; cat. n. 0030125150).
● 2 mL and 5 mL sterile and disposable serological pipettes (BD Falcon Becton Dickinson Labware, Franklin Lakes, NJ, USA; cat. n. 357507 and n. 357543, respectively).
● 15 mL conical, sterile, polypropylene, centrifuge tubes (Fisher Scientific, Pittsburgh, PA, USA; model Corning 352196).

#### 2.2.2 Step-by-step protocol

(1) Collect 8 mL of blood sample in an EDTA-tube (tube volume: 2 mL).

**▲ NOTE: For optimal cell viability, blood samples must be processed within 2 hours of collection.**

(2) Add Lymphosep to a 15 mL tube at a 1:2 ratio to blood. For best results, use 4 mL blood with 4 mL Lymphosep per tube, splitting 8 mL blood into two tubes. Avoid 50 mL tubes, as they reduce ring thickness at the interface.

**! Critical step: it is not necessary to dilute blood with D-PBS.**

**○ Comment: The 1.077 g/mL gradient separates blood into two fractions: PBMCs in the upper (low-density) layer, and RBCs plus PMNs in the lower (high-density) layer.**

(3) Slowly add blood onto the gradient by inclining the tube and pipetting dropwise along the tube wall to avoid mixing and ensure a clear interface.

**▲ NOTE: Set the pipettor speed at “low” or “zero” to facilitate the layering of blood on the interface.**

(4) Centrifuge at 1010×*g* for 20 minutes at room temperature with zero brake and acceleration to prevent phase mixing. After stopping, carefully remove the tube to avoid disturbing the layers.
(5) After centrifugation, PBMCs form a cloudy white layer in the middle. Plasma is above, and erythrocytes with granulocytes settle below. A clear interface indicates good separation.
(6) **! Critical step: The PBMC layer should be carefully aspirated using a 1000 μL pipette to minimize contamination and then transferred to a clean 15 mL tube for further processing.**

**○ Comment: The gradient solution can be toxic to cells, so complete this process quickly, especially if planning to culture the PBMCs after extraction**.

(7) Add D-PBS 1:1 (v/v). Mix gently and rapidly.
(8) Centrifuge sample at 605×*g* for 15 min at room temperature with rotor brake on.
(9) Discard the supernatant and replace with fresh D-PBS. The volume depends on the pellet size (use from 1 to 3 mL); gently resuspend the pellet.
(10) Centrifuge again at 300×*g* for 12 min at room temperature with rotor brake on.
(11) Discard the supernatant and resuspend cells in 1–2 mL RBC lysis buffer (depending on the pellet size), leave the pellet undisturbed for 15 min at room temperature to give time to the RBC lysis to disrupt the red blood cells and then centrifuge at 300×*g* for 15 min.

**▲ NOTE: This step is not necessary if the cell pellet is not contaminated by red blood cells.**

**○ Comment: RBC Lysis Buffer is formulated for optimal lysis of erythrocytes in single-cell suspensions of human peripheral blood. The buffers contain ammonium chloride, which lyses erythrocytes with minimal effect on leukocytes.**

(12) Resuspend cells in the appropriate amount of cell culture medium and count cells with trypan blue; or see the comments (optional).

**○ Comments: PBMCs can be analyzed by flow cytometry, cultured in RPMI with 10 % FBS or human serum, or stored at -80°C in 90 % serum and 10 % DMSO for later use.**

### 2.3 CTC isolation methods

We isolated circulating tumor cells (CTCs) using three methods: RosetteSep™ (Diagrammatic Representation 2A), MACS® (Diagrammatic Representation 2B), and Parsortix™ (Diagrammatic Representation 2C) systems. RosetteSep™ (Anti-CD36) enables rapid, column-free negative selection by binding unwanted cells to red blood cells, pelleting them during density gradient centrifugation, leaving unlabeled CTCs at the plasma interface with up to 98 % purity (www.stemcell.com/PIS). MACS® Tumor Cell Isolation Kit depletes tumor-associated cells without harming CTCs; we modified the protocol by omitting Non-Tumor Cell Depletion Cocktail B to preserve mesenchymal CTCs (Order no. 130-108-339, www.miltenyibiotec.com). Parsortix™ uses microfluidics to isolate CTCs based on size and deformability without antibodies (www.angleplc.com). For all methods, CTCs were resuspended in 200 μL RPMI with 10 % FBS, plated for immunofluorescence staining, or stored at −80 °C for DNA/RNA extraction.

### 2.4 Exosome isolation from PBMCs via a differential ultracentrifugation protocol

Ultracentrifugation is the gold standard for EVs isolation, using multiple spins to separate EVs by size and density while removing debris, dead cells, and proteins (Diagrammatic Representation 3A).

#### 2.4.1 Materials and equipment

● Centrifuge (Thermo Scientific, Cincinnati, OH, USA; model Heraeus Megafuge 1.0).
● Dulbecco's Phosphate buffered saline D-PBS (Gibco™, cat. n. 14190144).
● Membrane Filter, 0.22 μm pore size (MF-Millipore™, cat. n. GSWP04700).
● Pipettes (Eppendorf Research, Milan, Italy; n. 3123000047 (10–100 μL), n. 3123000063 (100–1000 μL), with relative sterile tips).
● Polyallomer tubes or polycarbonate bottles, appropriate for the ultracentrifuge rotor (Beckman Coulter, Brea, CA, USA).
● Ultracentrifuge and fixed- or swinging-bucket rotor.
● 1.5 mL tube (Eppendorf Research, Milan, Italy; cat. n. 0030125150).

#### 2.4.2 Step-by-step protocol

(1) Transfer the medium containing PBMCs into 15 mL centrifuge tubes.
(2) Centrifuge at 300×*g* for 12 min at 4 °C to pellet the cells.
(3) Collect the supernatant and transfer it into a new 15 mL centrifuge tube.
(4) Centrifuge at 2000×*g* for 10 min at 4 °C to remove dead cells.

Pipette off the supernatant, taking care not collect the pellet and transfer the EVs-containing medium to ultracentrifuge tubes appropriate for the ultracentrifugation rotor to be used.

**▲ NOTE**: Mark the tubes on one side and then place them in the rotor, ensuring that the mark is facing up. This allows for easy visualization of the pellet.

(5) Centrifuge for 30 min at 10,000×*g*, 4 °C.

**▲ NOTE**: Do not use the rotor brake to avoid interfering pellets.

(6) Collect the supernatant and filter it through a 0.22 μm filter to increase the purity of the sample.
(7) Transfer the filtered supernatant to a new ultracentrifuge tube.
(8) Centrifuge for 70 min at 100,000×*g*, 4 °C.

**○ Comment**: The EVs will not be damaged for a longer time (up to 3 hours) [20].

(9) Remove the supernatant.
(10) Resuspend the pellet with cold D-PBS.
(11) Perform an additional centrifuge for 70 min at 100,000×*g*, 4∗C to wash the EVs pellet and eliminate contaminating proteins.
(12) Remove the supernatant.
(13) Add 150–200 μL of cold D-PBS to resuspend the EVs.
(14) Transfer the EVs into 1.5 mL tubes and store at −80 °C for further use.

### 2.5 Isolation of RNA from TEPs

This methodology has been carefully described by previous authors, who developed an accurate strategy for isolation of good quality RNA from TEPs, in order to ultimately perform gene expression studies [7]. We adapted the protocol to study gene expression by nanoString™ methodology using an nCounter Analysis System (Diagrammatic Representation 3B).

#### 2.5.1 Materials and equipment

● Agilent 2200 TapeStation System (Agilent Technologies, CA, USA)
● Centrifuge (Thermo Scientific, Cincinnati, OH, USA; model Heraeus Megafuge 1.0).
● HEP buffer: 140 mM NaCl, 2.7 mM KCl, 3.8 mM HEPES, 5 mM EGTA, pH 7.4
● mirVana miRNA Isolation Kit (Ambion, cat. n. AM1560)
● nCounter® PanCancer Pathways Panel (nanoString, WA, USA)
● nCounter® Analysis Systems (nanoString, WA, USA).
● Pipettes (Eppendorf Research, Milan, Italy; n. 3123000047 (10–100 μL), n. 3123000063 (100–1000 μL), with relative sterile tips).
● Prostaglandin E1 (Santa Cruz Biotechnology, TX, USA. Cat n. sc-201223)
● The ROSALIND® Platform for nCounter® Analysis (nanoString, WA, USA).
● Wash buffer (10 mM sodium citrate, 150 mM NaCl, 1 mM EDTA, 1 % (w/v) dextrose, pH 7.4)

#### 2.5.2 Step-by-step protocol

(1) Collect whole blood in a tube containing EDTA and invert the tube 8–10 times
(2) Centrifuge the tubes at 200×*g* for 20 min at room temperature.
(3) Transfer the straw-colored platelet-enriched plasma (PRP) into a 15 mL tube
(4) Add HEP Buffer at 1:1 ratio
(5) Add Prostaglandin E1 (final concentration 1 μM)
(6) Centrifuge a 100×*g* for 20 min at room temperature to pellet contaminating red and white blood cells.
(7) Transfer the supernatant into a new 15 mL tube
(8) Centrifuge at 800×*g* for 20 min at room temperature.
(9) Rinse twice the platelet pellet with a wash buffer.
(10) Use the mirVana miRNA Isolation Kit following manufacturer instructions.
(11) Measure intact RNA concentration using the Agilent Tapestation 2200 Bioanalyzer
(12) Analysis of isolated RNA by NanoString PanCancer panel (770 genes) using the nCounter Analysis System.
(13) Gene expression analysis using the ROSALIND® Platform for nCounter® Analysis.

## 3 Results and discussion

### 3.1 Human peripheral blood mononuclear cells (PBMCs)

Biopsy tissues are valuable but hard to obtain and may cause patient discomfort. PBMCs are widely used for genomic studies due to easy, minimally invasive collection. However, PBMCs contain diverse immune cells, making gene expression interpretation complex due to variable subset proportions and inter-individual differences influenced by disease, treatment, isolation, processing, and cryopreservation methods. Standardizing PBMC isolation, culture, and storage protocols helps address these challenges.

**Fig. 1:**
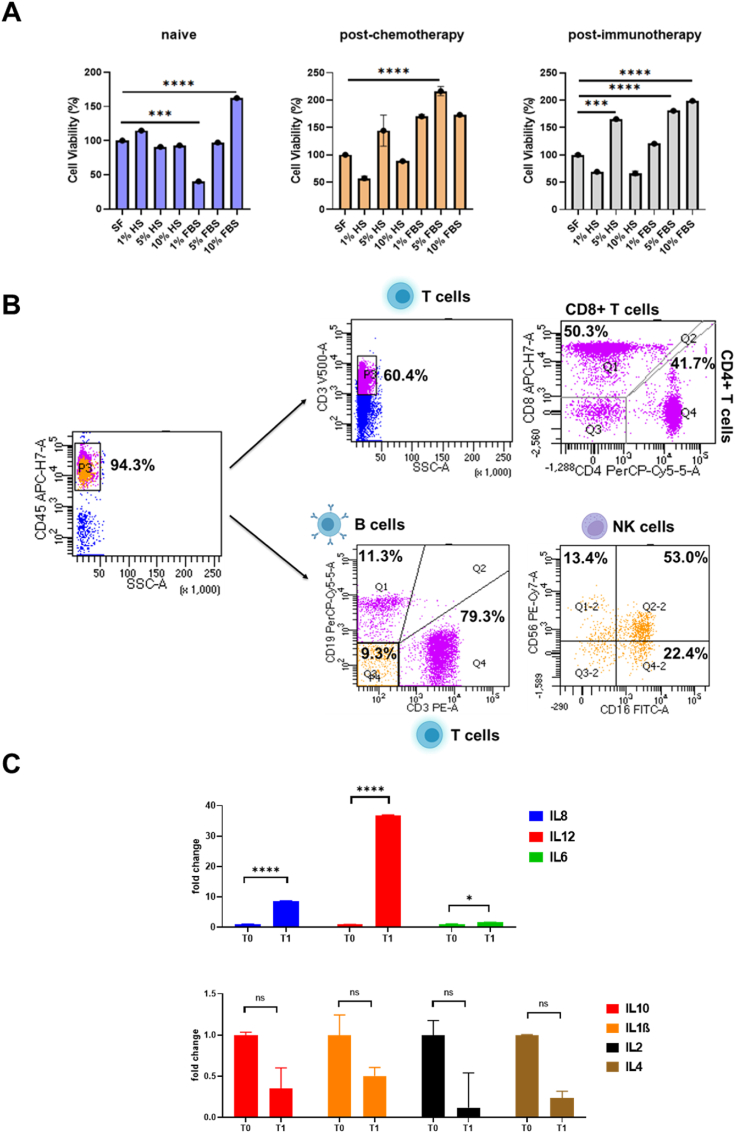
**(A)** Cell viability of the PBMCs from naive, post-chemotherapy, and post-immunotherapy patients after 24 h of culture in a serum-free medium or in a medium containing different percentages (1 %, 5 % and 10 %) of FBS or HS. Data are expressed as mean ± SD derived from n = 4 technical. One-tailed unpaired Student's t-test with CI = 95 %, ∗∗∗∗p < 0.0001, ∗∗∗p < 0.001. **(B)** FACS analysis of SCLC patients-derived PBMCs subset**. (C)** Real time PCR analysis of in vitro IL8, IL12, IL6, IL10, IL1β, IL2 and IL4 mRNA expression in SCLC patients-derived PBMCs collector before (T0) and after (T1) receiving chemotherapy treatment. Changes in mRNA levels were normalized to the expression of housekeeping genes (18s). Data are expressed as means ± SEM derived from n = 2 technically calculated by the comparative method 2-ΔΔCt. One-tailed unpaired Student's t-test with CI = 95 %, ∗∗∗∗p < 0.0001, ∗∗∗p < 0.001, ∗∗p < 0.05.

We isolated PBMCs from ES-SCLC patients—naive, post-cisplatin, and post-anti-PDL1 treatment—and cultured them in RPMI-1640 with varying fetal bovine serum (FBS) or human serum (HS) percentages to assess viability (Fig. 1). MTS assay after 24h showed 1 % FBS reduced viability, while 10 % FBS improved it in naive PBMCs. Post-chemotherapy PBMCs had decreased viability with 1 % and 10 % HS but increased with FBS. Post-immunotherapy PBMCs showed higher viability with 5 % HS and 5–10 % FBS. Overall, RPMI-1640 with 5–10 % FBS best maintains PBMC viability after 24h (Fig. 1A). Flow cytometry of gradient-isolated PBMCs cultured 24h in RPMI-1640 with 10 % FBS showed immune subsets: 60.4 % CD3^+^ T cells (50.3 % CD8^+^, 41.7 % CD4^+^), 11.4 % B cells, and 9.3 % NK cells (13.4 % CD56^+^, 22.4 % CD16^+^, 53.0 % CD56+CD16^+^) (Fig. 1B). This method preserves PBMC subset proportions. RT-PCR of PBMCs from SCLC patients before (T0) and after chemotherapy (T1) revealed significant increases in IL6, IL8, and IL12, indicating early treatment-induced immune changes detectable with this isolation protocol (Fig. 1C). In this respect, we previously demonstrated that changes in mRNA levels of innate immune sensors cGAS-STING downstream pathway chemokines such as CXCL10 and CCL5 as well as their circulating levels, may be related to tumor response to chemo-immunotherapy in lung cancer patients [4]. Overall, we conclude that using an efficient and standardized method to isolate and characterize PBMCs from blood patients may be helpful for monitoring the response to immunotherapy.

### 3.2 Circulating tumor cells (CTCs)

**Fig. 2:**
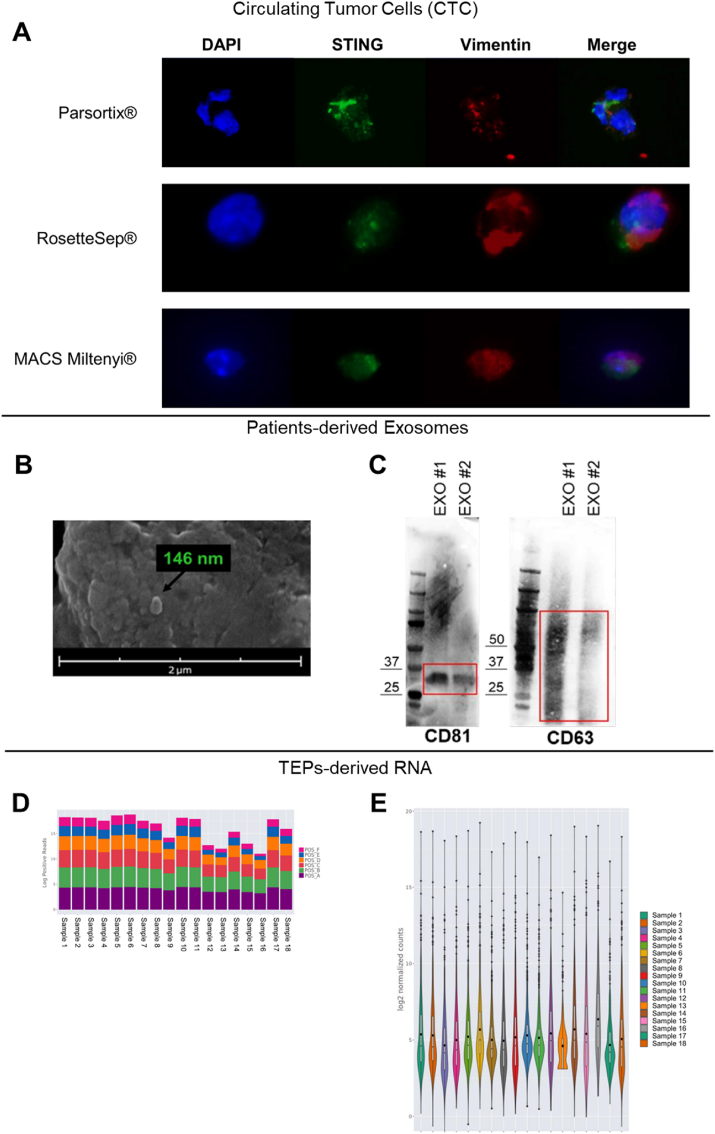
**(A)** Representative immunofluorescence images of CTCs with different extraction methods. Co-localization of STING (green, Alexa Fluor 488) and vimentin (red, Alexa Fluor 647). Nuclei were stained with DAPI (blue). Magnification 63X. **(B)** Representative SEM images of the isolated exosomes (scale bar = 2 μm). **(C)** Western blot of exosomal markers CD81 and CD63. The isolated exosomes with different protein concentrations (EXO#1 = 40 μg and EXO#2 = 20 μg) were successfully obtained from the culture supernatants of PBMCs from SCLC patients. Red line indicated the band of each protein on the gel. Original western blots are presented in File S1. **(D)** Stacked bar plots showing the log10-transformed positive control read counts across the samples. **(E)** Violin plot displaying the distribution of the log of the un-normalized gene counts for all the samples.

CTCs originate from solid tumors, entering blood directly or via lymphatics, and drive metastasis. They are extremely rare—1 to 10 per 10^6^–10^8^ white blood cells—and overlap with leukocytes in size and properties, requiring sensitive, high-purity isolation. Many lack EpCAM, and EMT markers like vimentin vary, complicating detection [8]. We applied three isolation methods to SCLC patient samples, staining CTCs for STING and vimentin to assess immune response and EMT features (Fig. 2A). All methods yielded high-purity CTCs, but Parsortix® provided the sharpest images and uniquely captured rare, highly metastatic CTC clusters. These clusters, which can detach intact from tumors and extravasate as groups, have greater metastatic potential and poorer prognosis than single CTCs, making their isolation critical for understanding metastasis and improving personalized cancer treatment.

**Diagrammatic Representation 1:**
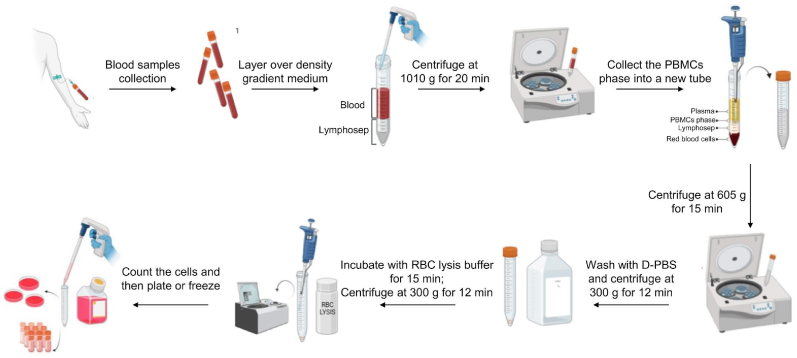
PBMCs isolation workflow using the density gradients method. Created with BioRender Tool. The graphical scheme was produced by the authors using the BioRender platform (https://www.biorender.com/) (basic licence terms).

**Diagrammatic Representation 2:**
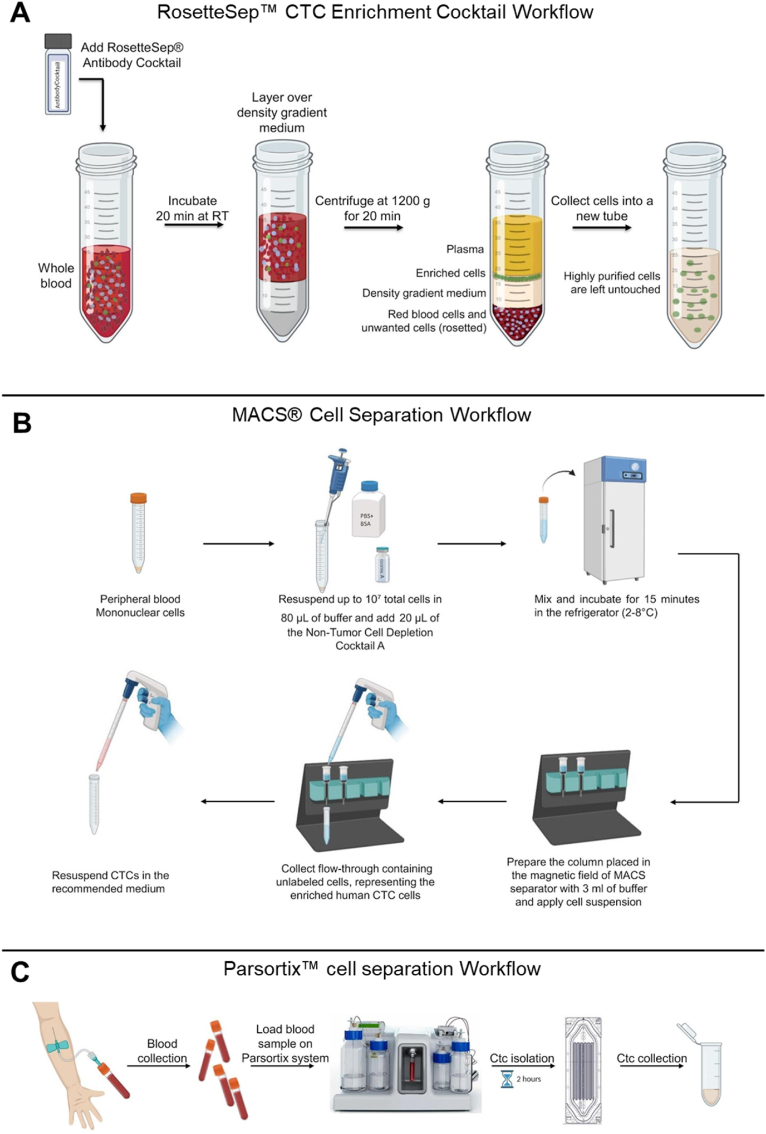
Protocol workflow for CTC isolation from whole blood using **(A)** RosetteSep™ CTC Enrichment Cocktail **(B)** MACS® Cell Separation technology **(C)** the Parsortix™ cell separation system. The graphical scheme was produced by the authors using the BioRender platform (https://www.biorender.com/) (basic licence terms).

**Diagrammatic Representation 3:**
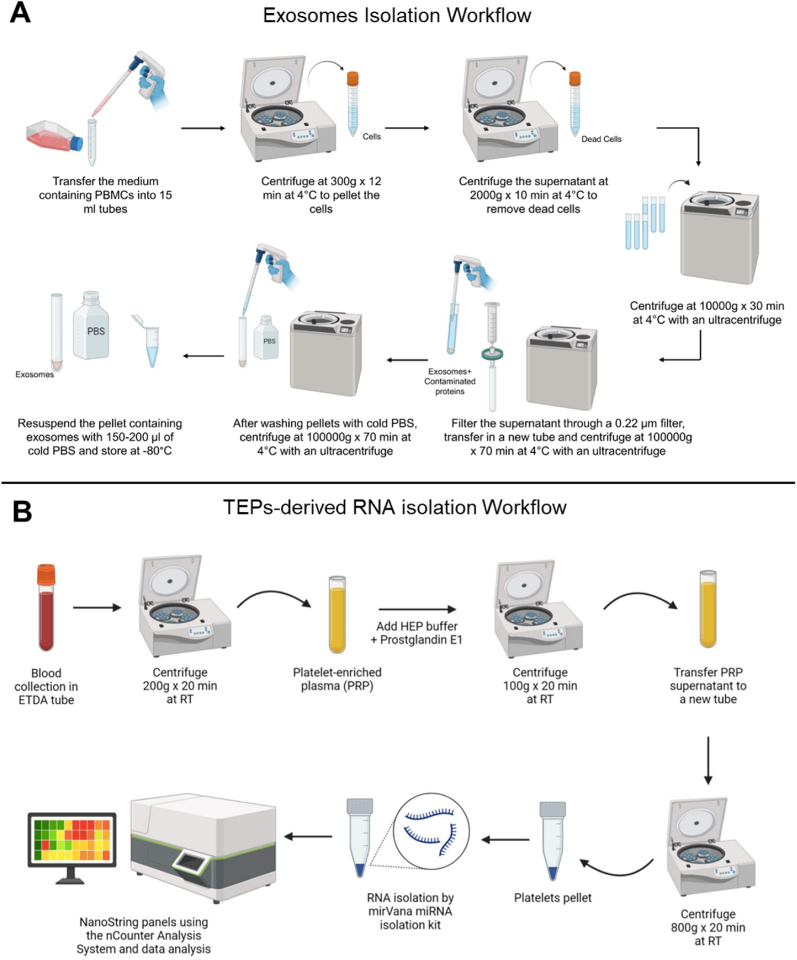
**(A)** Protocol workflow for EVs derived from PBMCs isolation using ultracentrifugation method. **(B)** Protocol workflow for RNA isolation from TEPs and analysis of gene expression by nCounter Analysis System. This figure was produced using the BioRender platform (https://www.biorender.com/) (basic licence terms).

### 3.3 Human circulating immune cells-derived EVs

Exosomes are 40–150 nm lipid vesicles released by most cells, circulating stably in body fluids like plasma, serum, CSF, urine, and culture media. Once seen as waste carriers, they are now recognized for intercellular communication, carrying bioactive molecules (nucleic acids, proteins, lipids) reflecting donor cell status. Common markers include tetraspanins (CD9, CD81, CD63, CD82), EMT, immune markers, glycoproteins, and adhesion molecules. These cargoes hold potential as cancer biomarkers for diagnosis, monitoring, and prognosis, supporting liquid biopsy use [9,10]. Our optimized PBMC-derived exosome isolation yields 20–40 μg protein, confirmed by FEG-SEM and Western blot (Fig. 2B–C), suggesting immune cell EVs as tools to study immune response in SCLC.

### 3.4 Isolation and analysis of TEPs-derived RNA

Tumor RNA analysis identifies cancer gene expression profiles [11]. While RNA extraction from frozen or FFPE tissues is established, liquid biopsy RNA isolation is challenging due to RNase contamination. We report preliminary quality control of RNA from liquid biopsies using Nanostring nCounter data analyzed with ROSALIND software. Quality checks included spike-ins, controls, housekeeping genes, and gene counts (Fig. 2D–E). Normalized reads were used for statistical analysis. Results confirm RNA extraction from TEPs is reliable, supporting its use as a non-invasive tool for diagnosis, monitoring, and research.

## 4 Discussion

Nowadays, LB is recognized as a powerful non-invasive approach to investigate changes of tumor heterogeneity over time. In the context of immuno-oncology, researchers are actively investigating blood-derived biomarkers that can predict response to immunotherapies, thereby personalizing treatment regimens for cancer patients, based on the identification of specific genetic alterations through circulating tumor DNA (ctDNA) [12]. However, there is an urgent need for standardized protocols for pre-analytical and analytical methods used in liquid biopsy to ensure consistent results across different laboratories and clinical trials for analysis of ctDNA, CTCs and ctRNA. The lack of standardized LB workflows poses a major barrier to clinical adoption. For example, CTC enumeration thresholds vary widely across studies (1–5 cells/7.5 mL blood), complicating their utility in prognostication. Similarly, EV isolation methods differentially enrich subsets (exosomes vs. ectosomes), confounding biomarker validation. Va.

To address this, we present a comprehensive framework to evaluate innovative blood-derived biomarkers—PBMCs, CTCs, immune cell-derived EVs, and TEP RNA—to improve cancer diagnosis and treatment. PBMCs reveal patient immune responsiveness and help identify predictive biomarkers, with IL dynamics offering insights into treatment effects. We developed three optimized CTC isolation protocols; notably, Parsortix preserves CTC structure, enhancing immunofluorescence staining and molecular characterization for deeper tumor biology understanding. We also adapted protocols to isolate circulating tumor RNA from TEPs for gene expression analysis. EVs, carrying protein cargoes reflecting their parent cells' state, provide tumor biology insights and therapeutic potential. Our prior work showed PBMC-derived exosomes exert cytotoxicity against SCLC cells, suggesting promising future applications. However, isolating CTCs and EVs remains technically contentious. For CTCs, EpCAM-based enrichment (CellSearch®) misses mesenchymal phenotypes, while label-free microfluidic approaches (e.g., Parsortix®) improve recovery but still lack scalability of samples. EV isolation is equally heterogeneous: differential centrifugation often co-pellets lipoproteins, whereas size-exclusion chromatography (qEV columns) or tangential flow filtration better preserve vesicle integrity. Non-blood fluids (e.g., cerebrospinal fluid, urine) present unique challenges; urinary EVs require additives like protease inhibitors to prevent degradation, while salivary EVs demand rapid processing to avoid bacterial contamination. Emerging techniques, such as acoustic sorting or EVClick® beads, may enhance specificity but require cross-platform validation. Therefore, to improve the reliability and reproducibility of liquid biopsy analyses, it is essential to implement comprehensive pre-analytical harmonization by adopting standardized operating procedures (SOPs) for blood collection, such as the use of PAXgene® ccfDNA tubes, strict processing timelines—ideally within 2 h for PBMC isolation—and appropriate storage conditions, including freezing samples at −80 °C in single-use aliquots [13]. Analytical benchmarking should be incorporated by utilizing spike-in controls, for example synthetic miRNAs for EVs RNA, alongside well-characterized reference materials like Horizon Discovery's ctDNA standards to calibrate and validate assay performance [14]. Additionally, data normalization strategies must be applied, including the systematic reporting of cfDNA fragment size distributions and protein markers such as CD63 and CD81 for EVs, to facilitate meaningful cross-study comparisons. Collaborative consortia, including BloodPAC, the International Society of Liquid Biopsy (ISLB) and the International Society for Extracellular Vesicles (ISEV), play a pivotal role in establishing these guidelines and ensuring that liquid biopsy technologies comply with regulatory frameworks such as IVDR and accreditation standards like CAP [[15], [16], [17]]. While emerging technologies, such as single CTC RNA sequencing and advanced EV tetraspanin profiling, hold promise for resolving biological heterogeneity, their successful translation into robust clinical tools depends fundamentally on rigorous standardization across all stages of sample handling and analysis.

## 5 Conclusions

Beyond the standard use of LBs, including the analysis of ctDNA, through the use of non-invasive methods for the analysis of PBMCs, CTCs, EVs and RNA from TEPs, it is possible to realize the full potential of LB technologies in translational cancer research, integrating data in LBs. Altogether, our study gives an improvement to the innovative blood-derived biomarkers methodology thus enhancing the LB field evolution towards more personalized cancer treatment strategies and ultimately improving patient outcomes.

## Informed consent statement

Informed consent was obtained from all subjects involved in this study.

## Institutional review board statement

This study was conducted in accordance with the Declaration of Helsinki and approved by the Ethics Committee of the University of Campania “Luigi Vanvitelli”, Naples (n. 280 on May 16, 2020).

## Data availability statement

Data will be made available on request.

## Funding

This work was supported by Fondazione AIRC (MFAG Project Number: 26237 to C.M.D.C.).

## Declaration of competing interest

Morgillo F.: receipt of honoraria or consultation fees for speaker, consultancy or advisory roles: Roche, Servier, Incyte, ESMO and MSD. Ciardiello F.: receipt of honoraria or consultation fees for speaker, consultancy or advisory roles: Amgen, Merck KGaA, MSD, Pierre Fabre, Pfizer, Roche and Servier; institutional financial interests, financial support for clinical trials or contracted research: Amgen, Merck KGaA, MSD, Pierre Fabre, Pfizer, Roche and Servier. Servetto A: receipt honoraria or consultation fees for speaker or advisory board from: AstraZeneca, ESMO, MSD, Bristol-Myers Squibb, Gilead, Eli Lilly, Roche, Regeneron, Novartis, Johnson&Johnson, outside the submitted work. Della Corte C.M.: reported receiving personal fees and travel grants from Roche, MSD, Novartis, Lilly, Regeneron, Amgen, Merck, Pfizer and AstraZeneca outside the submitted work. The remaining authors have no conflicts of interest to declare.

## References

[1] Corte C.M.D., Cimmino F., Morgillo F. (2021). Analysis of DNA from liquid biopsy: new genetic biomarkers for cancer immunotherapy?. Explor Target Antitumor Ther.

[2] De Rosa V., Fonti R., Vecchio S.D., Iommelli F. (2021). Non-invasive detection of epithelial mesenchymal transition phenotype and metastatic dissemination of lung cancer by liquid biopsy. Explor Target Antitumor Ther.

[3] Alix-Panabières C., Pantel K. (2021). Liquid biopsy: from discovery to clinical implementation. Mol Oncol.

[4] De Rosa C., Iommelli F., De Rosa V., Ercolano G., Sodano F., Tuccillo C., Amato L., Tirino V., Ariano A., Cimmino F., di Guida G., Filosa G., di Liello A., Ciardiello D., Martinelli E., Troiani T., Napolitano S., Martini G., Ciardiello F., Papaccio F., Morgillo F., Della Corte C.M. (2024). PBMCs as tool for identification of novel immunotherapy biomarkers in lung cancer. Biomedicines.

[5] Antunes-Ferreira M., Koppers-Lalic D., Würdinger T. (2021). Circulating platelets as liquid biopsy sources for cancer detection. Mol Oncol.

[6] Sheikhhossein H.H., Iommelli F., Di Pietro N., Curia M.C., Piattelli A., Palumbo R., Roviello G.N., De Rosa V. (2024). Exosome-like systems: from therapies to vaccination for cancer treatment and prevention-exploring the state of the art. Vaccines (Basel).

[7] Neumann M.H.D., Bender S., Krahn T., Schlange T. (2018). ctDNA and CTCs in liquid biopsy - current status and where we need to progress. Comput Struct Biotechnol J.

[8] Wang Zhihui (2025).

[9] Keller S., Ridinger J., Rupp A.K., Janssen J.W., Altevogt P. (2011). Body fluid derived exosomes as a novel template for clinical diagnostics. J Transl Med.

[10] Coughlan C., Bruce K.D., Burgy O., Boyd T.D., Michel C.R., Garcia-Perez J.E., Adame V., Anton P., Bettcher B.M., Chial H.J., Königshoff M., Hsieh E.W.Y., Graner M., Potter H. (2020). Exosome isolation by ultracentrifugation and precipitation and techniques for downstream analyses. Curr Protoc Cell Biol.

[11] Best M.G., In 't Veld S.G.J.G., Sol N., Wurdinger T. (2019). RNA sequencing and swarm intelligence-enhanced classification algorithm development for blood-based disease diagnostics using spliced blood platelet RNA. Nat Protoc.

[12] Amato L., De Rosa C., De Rosa V., Heydari Sheikhhossein H., Ariano A., Franco P., Nele V., Capaldo S., Di Guida G., Sepe F., Di Liello A., De Rosa G., Tuccillo C., Gambardella A., Ciardiello F., Morgillo F., Tirino V., Della Corte C.M., Iommelli F., Vicidomini G. (2024). Immune-cell-derived exosomes as a potential novel tool to investigate immune responsiveness in SCLC patients: a proof-of-concept study. Cancers (Basel).

[13] Greytak Sarah R. (2020). Harmonizing cell-free DNA collection and processing practices through evidence-based guidance. Clin Cancer Res.

[14] De Luca, Thomas (2021). Novel quantification of extracellular vesicles with unaltered surface membranes using an internalized oligonucleotide tracer and applied pharmacokinetic multiple compartment modeling. Pharm Res.

[15] Leiman Lauren C. (2022). Creating standards for liquid biopsies: the BLOODPAC experience. Expert Rev Mol Diagn.

[16] Fusco Nicola (2025). Role of the international society of liquid biopsy (ISLB) in establishing quality control frameworks for clinical integration. Crit Rev Oncol Hematol.

[17] Royo Felix (2020). Methods for separation and characterization of extracellular vesicles: results of a worldwide survey performed by the ISEV rigor and standardization subcommittee. Cells.

