# Supplementary material for: Novel applications of liquid Biopsy: Comprehensive methodology for circulating biomarker exploration in peripheral blood
Source: J Liq Biopsy. 2025 Jun 26;9:100307. doi: 10.1016/j.jlb.2025.100307 (PMC12272590; doi:10.1016/j.jlb.2025.100307)
Supplement: Multimedia component 1 [file mmc1.pdf]

S1. Supplementary methods

S1.1. MTS assay

After isolating the PBMCs from naive, post-chemotherapy, and post-immunotherapy patients, the viability of the PBMCs after 24 hours of culturing was assessed using MTS assay. Briefly, PBMCs were seeded in 96-well flat-bottomed plates at a density of 5000 cells/well and cultured in a serum-free medium or in a medium with different percentages (1%, 5% and 10%) of fetal bovine serum (FBS) or autologous human serum (HS). Following a 24 h incubation period, the MTS reagent (MTS Assay Kit, cat. no. ab197010, Abcam) was added (10 µL/well) to assess the reduction of the MTS tetrazolium compound by viable cells, resulting in the generation of a coloured formazan dye that is soluble in cell culture media. The conversion is conducted by NAD(P)H-dependent dehydrogenase enzymes in metabolically active cells. The samples were incubated with MTS reagent for 3 hours under standard culture conditions. Subsequently, the plate was shaken briefly and the absorbance measured spectrophotometrically at 490 nm (Tecan). The number of viable cells was expressed as the percentage of viable cells, considering the untreated control cells as 100%. At least three independent experiments were performed in triplicate.

S.1.2. Flow Cytometry

After isolation, PBMCs from small cell lung cancer (SCLC) patients were stained and analysed using standard flow cytometry approaches. Single cell suspensions from all samples were washed with FACS buffer (1% FBS in PBS) and then stained with cell surface marker antibodies for 45 min at 4 °C. Antibodies used were APC-Cy7 Mouse Anti-Human CD45 (clone 2D1) (cat. n 561863, BD Biosciences), V500 Mouse Anti-Human CD3 (clone UCHT1) (cat. n. 561417, BD Biosciences), PerCP-Cy5.5 Mouse Anti-Human CD4 (clone SK3) (cat. n. 566316, BD Biosciences), APC-H7 Mouse Anti-Human CD8 (clone SK1) (cat. n. 560179), PE Anti-human CD3 (clone SK7) (cat. n. 340662, BD Biosciences), PE Mouse Anti-Human CD56 (NCAM-1) (cat. n. 555516, BD Biosciences), PerCP-Cy5.5 Mouse Anti-Human CD19 (clone SJ25C1) (cat. n. 340951, BD Biosciences) and FITC Mouse Anti-Human CD16 (3G8) (cat. n. 556618, BD Biosciences). Labelled cells were washed and resuspended in FACS buffer for flow cytometric analysis on a BD Fortessa with FACS Diva software (BD Biosciences, Franklin Lakes, NJ, USA) and analysed using FACS Diva software, version 8.0. Negative gating was based on a fluorescence-minus-one (FMO) strategy.

S.1.3. RNA Extraction, cDNA synthesis and qRT-PCR

Total RNA was obtained from PBMCs using TRIzol™ Reagent (Invitrogen™, Santa Clara, CA, USA; cat. n. 15596026), according to the manufacturer's protocol. Briefly, PBMCs were lysed with 1 mL of TRIzol and the lysates were vortexed to improve homogenisation. Incubate the Eppendorf tubes for 5 min at RT. Chloroform (0.2 mL) was added and samples were shaken vigorously by hand for 15 sec and incubated for 3 min at RT. Samples were then centrifuged at 12,000× *g* for 15 min at 4 °C. The mixture separates into a lower red phenol-chloroform, and interphase, and a colourless upper aqueous phase. The aqueous phase was removed for RNA extraction and transferred to another tube. RNA was precipitated by mixing with cold isopropyl alcohol (0.5 mL) for 2 h at −80 °C. Samples were centrifuged at 12,000× *g* for 10 min at 4 °C and washed twice with 75% ethanol. Total RNA was suspended in 50 µL of 0.1 mM EDTA-RNase-free water and then incubate in a water bath or heat block set at 55–60°C for 10–15 minutes. The purity and concentration of RNA were determined by OD 260/280 readings using the Nanodrop 2000 spectrophotometer (Thermo Fisher Scientific, Waltham, MA, USA). After RNA extraction, cDNA was generated from 500 ng of total RNA using a SensiFAST cDNA Synthesis Kit (BIO-65053, Meridian Bioscience, Memphis, TN, USA) under the following conditions: 25 °C for 10 min, 42 °C for 15 min, 85 °C for 5 min. mRNA expression levels of different genes were evaluated by qRT-PCR with a QuantStudio 7-Flex (Applied Biosystems by Life Technologies, Monza, Italy) using the SensiFAST SYBR Hi-ROX Kit (BIO-92005, Meridian Bioscience, Memphis, TN, USA) under the following conditions: 50 °C for 2 min (stage 1) followed by a denaturation step at 95 °C for 10 min (stage 2) and then 40 cycles at 95 °C for 15 s and 60 °C for 1 min (stage 3). All samples were run in duplicate, in 20 µL reactions and relative expression of genes was determined by normalising to *18S*, used as an internal control gene; to calculate relative gene expression in value we used the $2^{-\Delta Ct}$ or $2^{-\Delta\Delta Ct}$ method. The list of primer sequences used for qRT-PCR is shown in Table 1.

Gene	Forward Sequence	Reverse Sequence
18S	*5′-CGCCGCTAGAGGTGAAATTC-3′*	*3′-CTTTCGCTCTGGTCCGTCTT-5′*
IL8	*5′-CTGTTAAATCTGGCAACCCTAGTCT-3′*	*3′-CAAGGCACAGTGGAACAAGGA-3′*
IL12	*5′-TGCCTTCACCACTCCCAAAACC-3′*	*3′-CAATCTCTTCAGAAGTGCAAGGG-5′*
IL6	*5′-AGACAGCCACTCACCTCTTCAG-3′*	*3′-TTCTGCCAGTGCCTCTTTGCTG -5′*
IL10	*5′-TCTCCGAGATGCCTTCAGCAGA-3′*	*3′-TCAGACAAGGCTTGGCAACCCA-5′*
IL2	*5′-AGAACTCAAACCTCTGGAGGAAG-3′*	*3′-GCTGTCTCATCAGCATATTCACAC-5′*
IL4	*5′-CCGTAACAGACATCTTTGCTGCC-3′*	*3′- GAGTGTCCTTCTCATGGTGGCT-5′*
IL1β	*5′-CCACAGACCTTCCAGGAGAATG-3′*	*3′-GTGCAGTTCAGTGATCGTACAGG-5′*

S.1.4. Immunofluorescence

Once the CTCs have been recovered by one of the previously described methods, the next step is to proceed with immunofluorescence. The CTCs were first resuspended in 200 µl of PBS, and the resulting cell suspension was dispensed onto a SBS slide (Tethis S.p.A.). The slide was incubated at room temperature to allow for adhesion. Following a period of 20 min, the PBS is aspirated from the slide, and three further washes are performed utilising PBS. The cells were then fixed for 20 min with a 4% paraformaldehyde (PFA) solution and permeabilized for 30 min with 0.3% Triton X-100 1% BSA in phosphate-buffered saline (PBS) at room temperature. Three additional washes with PBS followed by incubation overnight at 4°C with the primary antibody Sting (D2P2F) (Cell Signalling, cat. n. 13647, 1:800) and Vimentin (Santa Cruz, cat. n. sc-6260, 1:200) in antibody dilution buffer (0.1% Triton X-100 in 1% BSA PBS) followed by revelation using Alexa Fluor 488-conjugated anti-rabbit IgG antibodies (Jackson Immunoresearch Laboratories, West Grove, PA, USA) and Alexa Fluor 647-conjugated anti-mouse IgG antibodies (Jackson Immunoresearch Laboratories, West Grove, PA, USA) at a dilution of 1: 250 for 1 h. Nuclei were stained with DAPI (1µg/mL) (Sigma). The fluorescence was analysed by an APOTOME microscope.

S.1.5. Scanning Electron Microscopy (SEM)

Pellets containing PBMC-EXs were resuspended in 0.1 – 0.4 mL of PBS, and a few microliters of suspension were deposited on a cover glass and dried overnight. The samples were positioned on a stub, directly transferred to an SEM (FEG SEM model Pharos from Thermo Fisher Scientific, Waltham, MA, USA) and subsequently sputter coated with an automatic sputter (Luxor Pt coater model, APTCO TECHNOLOGIES, Nazareth, Belgium) using gold in air and setting a thickness of approximately 5 nm. The samples were then analysed in a high vacuum using an SED (secondary electron) detector at an accelerating voltage of 15 kV. At least 3 EM images were acquired for each sample.

S.1.6. Western Blot

PBMCs were lysed by homogenization in an RIPA lysis buffer [0.1% sodium dodecyl sulphate (SDS), 0.5% deoxycholate, 1% Nonidet, 100 mmol/L NaCl, 10 mmol/L Tris–HCl (pH 7.4), 0.5 mmol/L dithiothreitol and 0.5% phenylmethyl sulfonyl fluoride], protease inhibitor cocktail (Hoffmann-La Roche, Basel, Switzerland)

and phosphatase inhibitor tablets (PhosSTOP; Roche Diagnostics, Basel, Switzerland) and clarification by centrifugation at 12500 rpm for 20 min at 4◦C. Whole cell lysates containing comparable amounts of proteins were resuspended in LDS reducing sample buffer, mixed and boiled at 100◦C for 10′ . Samples were resolved by SDS-PAGE gels and electro-transferred onto 0.2 µm nitrocellulose membranes (Trans-Blot Turbo; BioRad). After blocking membranes for 90 min at room temperature, they were incubated overnight at 4◦C with primary antibodies and then with a secondary antibody for 1 h at room temperature. Horseradish peroxidase-linked anti-rabbit (BioRad) and anti-mouse (BioRad) antibodies were used as secondary antibodies. Proteins were detected with a Clarity Western ECL Substrate using the ChemiDoc system (BioRad). Images were analysed using BioRad software Image Lab 3.0.1. Monoclonal anti-CD81 (sc166029) and anti-CD63 (sc-15363) antibodies were obtained from Santa Cruz Biotechnology, Dallas, Germany (Amato, Luisa, et al. "Immune-Cell-Derived Exosomes as a Potential Novel Tool to Investigate Immune Responsiveness in SCLC Patients: A Proof-of-Concept Study." Cancers 16.18 (2024): 3151.).
