# Supplementary figures and images for: Novel applications of liquid Biopsy: Comprehensive methodology for circulating biomarker exploration in peripheral blood
Source: J Liq Biopsy. 2025 Jun 26;9:100307. doi: 10.1016/j.jlb.2025.100307 (PMC12272590; doi:10.1016/j.jlb.2025.100307)

Uncropped gels Figure 2B

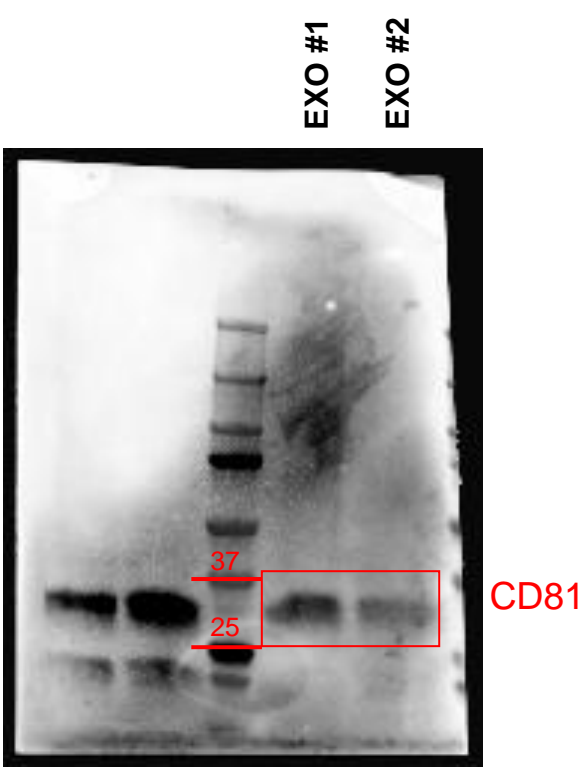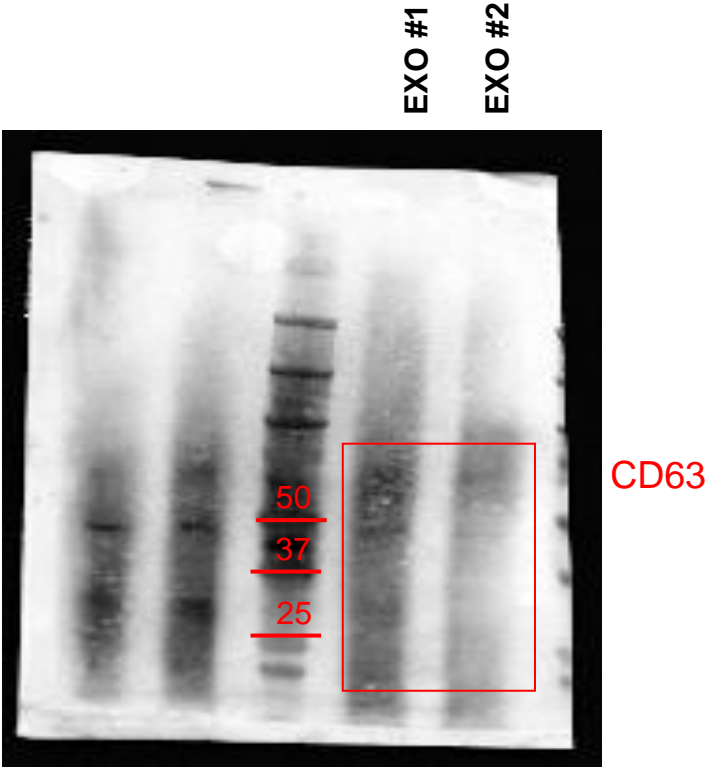

Supplement: Multimedia component 2 [file mmc2.pdf]
